# Long-term follow-up of metatropic dysplasia caused by novel mutations in the TRPV4 gene: Case report and literature review

**DOI:** 10.1097/MD.0000000000042034

**Published:** 2025-04-18

**Authors:** Yu Liu, Yanzhao Dong, Junfang Xu, Bing Xia, Weiming Hu, Xinwei Li, Feipeng Wang, Yufeng Zhao, Guoming Feng

**Affiliations:** a Department of Orthopedics, The Third Affiliated Hospital of Zhengzhou University, Zhengzhou, China.

**Keywords:** case report, genotype–phenotype, metatropic dysplasia, skeletal dysplasia, Transient Receptor Potential Vanilloid 4 gene

## Abstract

**Rationale:**

To observe the natural history of the disease and the radiographic evolution of growth and development in patients with metatropic dysplasia (MD) and to complement the spectrum of mutations in the transient receptor potential vanilloid 4 (TRPV4) gene and the spectrum of MD phenotypes.

**Patient concerns:**

We report a patient with MD caused by a novel missense mutation in TRPV4, who possessed a mixed phenotype of both abnormal skeletal development and peripheral neuropathy. From 3 months to the age of 7 years, we observed the patient’s natural history and the imaging evolution of the patient’s growth and development.

**Diagnosis:**

The diagnosis of MD based on growth and developmental history, clinical presentation, imaging and mutation analysis of the TRPV4 gene.

**Interventions:**

She underwent posterior spinal osteotomy (T10, vertebral column resection), lateral kyphosis correction, internal fixation (T6-L3), and implant fusion. Surgical intervention can effectively delay the course of the disease.

**Outcomes:**

Sequencing analysis and family validation of the patient’s whole exon gene confirmed for the first time that the mutation in exon 11 of the TRPV4 gene was a heterozygous missense mutation (c.1811T > A) resulting in the mutation of isoleucine at position 604 to asparagine (p. I604N).

**Lessons:**

This study complements the spectrum of mutations in the TRPV4 gene and the spectrum of MD phenotypes and provides a reference for prenatal diagnosis, genetic counseling, mechanistic studies, and development of symptomatic treatment for this type of disease.

## 1. Introduction

Metatropic dysplasia (MD) is a rare condition of congenital skeletal dysplasia first described by Maroteaux et al^[1]^ and is based on changes in body proportions after birth and during childhood. It is distinguished by thoracic constriction, shorter limbs, protruding and limited joints, progressively deteriorating lateral and kyphosis of the spine, and sometimes caudal appendages, as well as joint contracture abnormalities.^[2,3]^ Long bones that are shaped like a dumbbell, flattened vertebrae, short ribs, broad flattened iliac bones, and expanded long bone epiphyses are radiological characteristics.^[2]^ While the severity of the MD phenotype can vary greatly and can be classified as mild, classic, or lethal from a clinical perspective, distinctions between the subtypes are not always obvious.^[1,4]^

MD is found only in the transient receptor potential vanilloid 4 (TRPV4) gene variants, an autosomal dominant disorder caused by heterozygous pathogenic variants in the TRPV4 gene.^[5]^ TRPV4 gene variations can cause chondrocyte overproduction, disrupt chondrocyte maturation, and impede endochondral ossification, all of which can result in a variety of skeletal system disorders, the most serious of which is MD.^[6–8]^ Mutations in the TRPV4 gene not only lead to abnormal development of the skeletal system, but also to peripheral neuromuscular disorders.^[9]^ Affected individuals usually have only neuromuscular or skeletal manifestations, with just a few cases having a combined phenotype of skeletal abnormalities and neuromuscular pathology.^[10]^ A few individuals have both skeletal abnormalities and neuromuscular pathology.

This study reports the natural history of a patient with MD from 3 months to 7 years of age who developed a neuromuscular pathology phenotype during growth and development, and gene sequencing confirmed a de novo mutation site on exon 11 of the TRPV4 gene. The aim of this case was to complement the spectrum of mutations in the TRPV4 gene and the spectrum of MD phenotypes. It was studied in the context of the clinical phenotype and imaging features, and the relevant literature reports were reviewed to improve the understanding and diagnosis of this disease.

## 2. Case presentation

**Figure 1.**
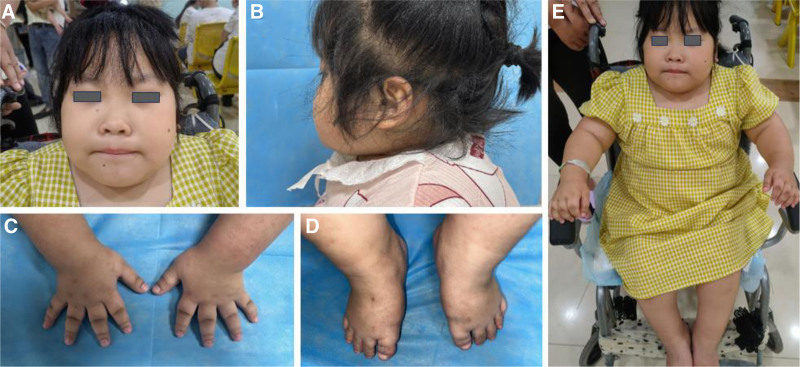
External view of the patient: (A) flat facial profile, low nose, square chin, duckbill. (B) Protruding jaw, short neck. (C and D) Short fingers and toes with extra creases. (E) The complete photos of the body.

The patient, a female 7-year-old, was admitted to our hospital because of abnormal skeletal development for 6 years and incomplete paralysis of both lower limbs for more than 3 months. Physical examination: height: 110 cm, weight: 60 kg, normal intellectual development, flat facial profile, low nose, square chin, protruding jaw, short neck, duckbill, pygmy body type, short and stubby fingers and toes with superfluous folds (Fig. 1), scoliosis and kyphosis, as well as coarse limb joints, there were several joint contractures, particularly in the hip, knee, and ankle joints; bilateral knee valgus; muscle strength of both lower limbs was grade 1, and the patient was unable to stand. The muscle strength of both upper limbs is about grades 3 to 4. She had no problems with urination, breathing or vocalization, and an MRI of the spine showed no spinal cord injury or cauda equina compression.

**Figure 2.**
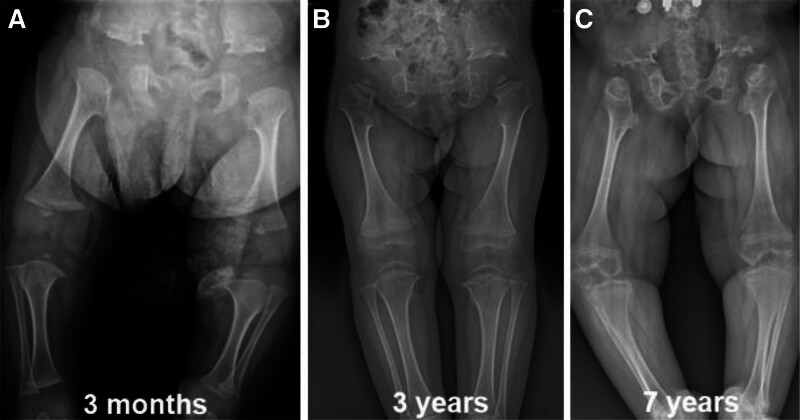
Imaging evolution of the long bones of the lower limbs: (A–C) hip dislocation on both sides, short and thick femur and tibiofibula on both sides, dilated long bone epiphyses with dumbbell shape, short and wide iliacs, shallow and flat acetabulums, supraspinatus cuts, and short and tilted femoral necks on both sides, while the length of the long bones significantly increased over time.

**Figure 3.**
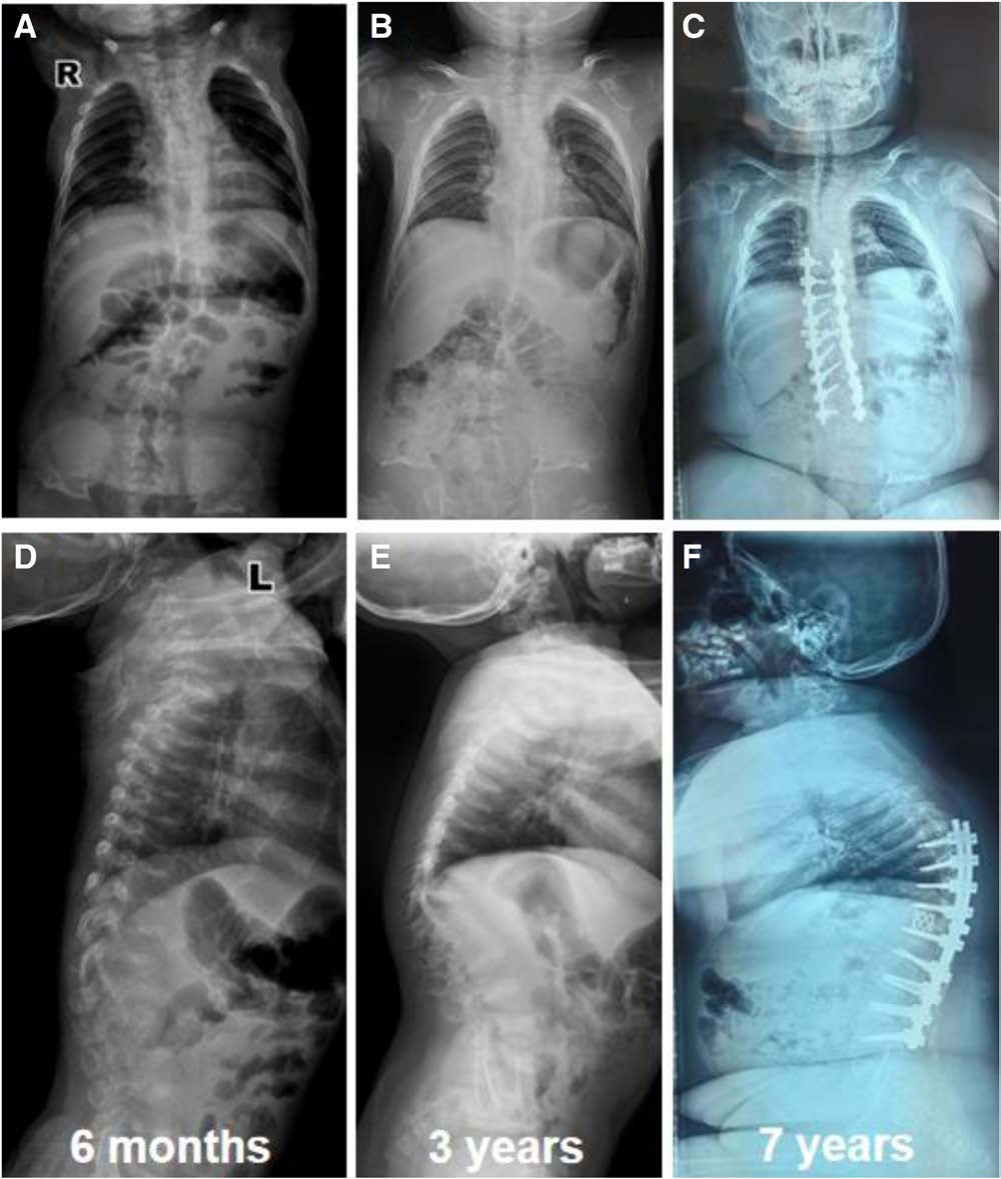
Imaging evolution of the full spine: (A–C): anteroposterior view of the spine: narrowing of the thorax, progressive worsening of the scoliosis, flattening and widening of the ribs bilaterally, good correction of the scoliosis at 2 years postoperatively. (D–F) Lateral view of the spine: the vertebral body flattened and thinned, and with time the vertebral body was almost near normal, and the kyphosis gradually worsened again with growth and development 2 years after the operation.

The patient was born by cesarean section due to fetal malposition and fetal cardiac instability, length at birth: 55 cm, weight 3550 g. She cried after birth and was found to have a bony bump in the sacrococcygeal region after birth, her head was straight up at 3 months of age, and she was unable to roll over, her chest was narrow and long, and her limbs were short and small (Fig. 2). From the age of 6 months, the joints and spine developed abnormally, manifesting as lateral and kyphosis of the spine, which was progressively aggravated (Fig. 3). The child was able to learn to walk, with multiple joint contractures in a duck-step at the age of 1 year and 6 months, and suffered bilateral hip dislocations at the age of 2 years, which were repaired by hip joint casts and endosteum release. She was able to walk on her own at the age of 4 years old despite a significant deterioration in the strength of her lower limb muscles, which was particularly noticeable in the joints of those extremities. At the age of 5 years, due to spinal deformity and incomplete paralysis of both lower limbs (ASIA grade D), she underwent posterior spinal osteotomy (T10, vertebral column resection), lateral kyphosis correction, internal fixation (T6-L3), and implant fusion. She was able to walk with the use of braces at the age of 6 years, and the progression of lateral kyphosis was halted.

**Figure 4.**
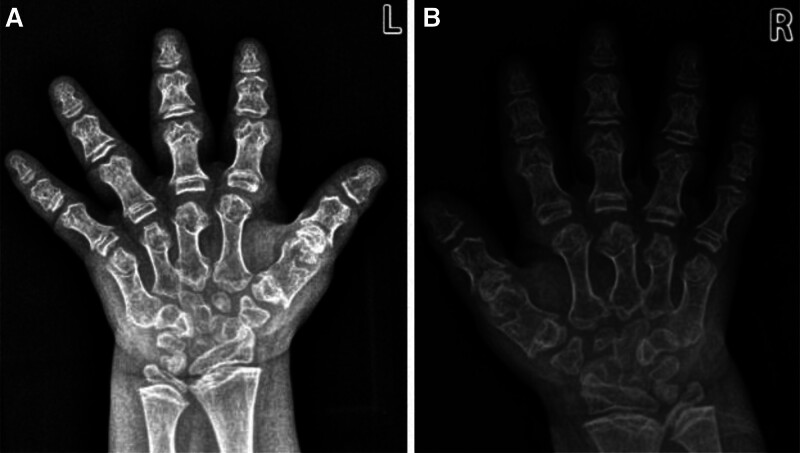
Imaging of both hands: (A and B) shortness of the metacarpophalangeal bones and enlargement of the metacarpophalangeal and ulnar-radial epiphyses in both hands.

Radiographic examination: the orthopantomogram of both lower limbs revealed the following characteristics: shallow and flat acetabulum, supra-acetabular notch, short and oblique neck of the femur, and extended epiphyses of all bones in the shape of a dumbbell (Fig. 2A–C). Full-length orthostatic view of the spine showed: thoracic stenosis, flattened and wide ribs, lateral kyphosis deformity of the spine, thinning and flattening of the vertebral bodies (Fig. 3A–F). Orthostatic position of both hands: short and thick metacarpals with expanded articular ends. The multiple small, irregular carpal bones are due to the delayed carpal ossification. The delayed carpal ossification is an imaging finding of MD (Fig. 4A and B).

**Figure 5.**
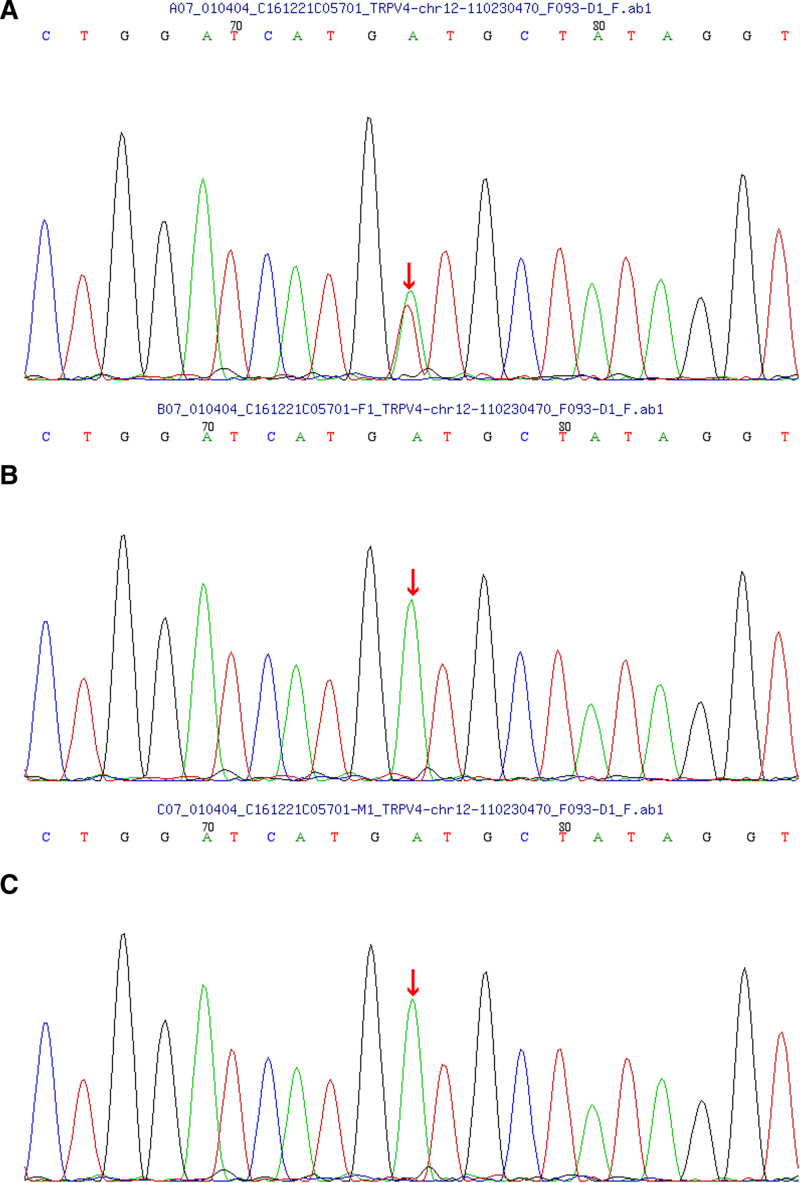
Validation of TRPV4 mutant gene family lines. (A) Heterozygous mutation in exon 11 of chromosome 12, c.1811T > A, as revealed by sequencing of the girl’s genome. (B) No mutation was found in her father. (C) No mutation was found in her mother.

Gene sequencing: skeletal gene check (MyGenostics): 1 heterozygous mutation was analyzed in the TRPV4 gene, c.1811T > A (nucleotide 1811 in the coding region was mutated from thymine to adenine), resulting in the amino acid change p.I604N (amino acid codon 604 was mutated from isoleucine to asparagine), a missense mutation. The examiner had no variation at this locus in this parent, so this mutation was spontaneous (Fig. 5).

## 3. Discussion

**Figure 6.**
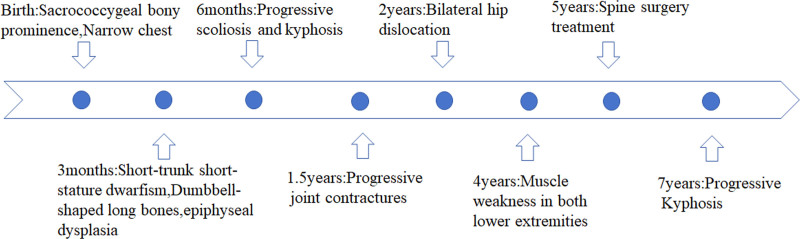
The course of MD progression. MD = metatropic dysplasia.

In this report, we describe a case of MD caused by a novel missense mutation in TRPV4, with long-term follow-up of the patient’s natural history from 3 months to 7 years of age (Fig. 6). The patient was born with short limbs and a narrow trunk, and during time, the lateral kyphosis got worse. However, the size of the long bones significantly improved, and the vertebrae nearly returned to normal (Figs. 1 and 2), and a gradual evolution to a short-trunked phenotype with significant changes in stature proportions (reversal), which corresponds to the description of autosomal dominant MD.

The TRPV4 gene is located on chromosome 12, q24.11, and contains 15 exons encoding TRPV4 protein, a polypeptide with 871 amino acid residues, containing an N-terminal proline-rich region, 6 ankyrin repeats domain, 6 transmembrane domains, and a C-terminal calmodulin binding domain. TRPV4 protein is widely distributed in bone, nerves, heart, blood vessels, skin and other tissues, and the N-terminal and C-terminal of the protein are located on the cytoplasmic side to sense a variety of regulatory factors,^[11,12]^ which are involved in the regulation of osmotic pressure sensing, cell volume maintenance, cell permeability regulation, pain-sensing and other important physiological processes.^[13,14]^

The specific functional domains in which the TRPV4 gene variants are located and the severity of the disease phenotype do not show a clear correlation, which makes it difficult to analyze the genotype–phenotype correlation of the TRPV4 gene, and the intrinsic relationship between the gene and the clinical phenotype is not clear. The current study suggests that MD severity is correlated with the degree of activation of TRPV4.^[7]^ The structural domain of calmodulin plays a very important role in the activity of this protein, and intracellular calcium ions can be bound to this region via Ca^2+^–CaM, thus increasing the activity of TRPV4 protein.

TRPV4 plays an important role in intracellular calcium channel ion regulation, chondrocyte differentiation, and osteoblast terminal differentiation. Gain-of-function (GOF) mutations in the TRPV4 gene lead to a range of musculoskeletal disorders, while missense variations dominate the TRPV4 gene mutation spectrum. The skeletal phenotypes associated with MD are mainly caused by GOF mutations in the TRPV4 gene, which not only activate calcium channels and lead to dysregulation of intracellular Ca^2+^ ion levels, but also up-regulate SOX9 expression and lead to abnormal chondrocyte differentiation.^[15,16]^ Han et al^[17]^ showed that the GOF variant in the TRPV4 gene disrupted osteoblast differentiation by increasing the activity of the Ca^2+^/NFATc1 pathway and induced the endochondral ossification disorders associated with MD. The GOF variant in the TRPV4 gene also resulted in increased bone turnover and decreased bone mass, which significantly increased the risk of osteoporosis and bone fractures, known as insufficiency fractures, which mainly occur in the metaphysis of the long bones.^[18]^

It is clinically difficult to clearly distinguish between the various forms of skeletal dysplasia caused by mutations in the TRPV4 gene, but this can be achieved by a combination of gene sequencing, clinical phenotyping and radiological criteria. The primary characteristic of MD is the progression of body proportions with age. A small chest, prominent joints, and a caudal tail are clinical characteristics of MD, however similar characteristics are occasionally present in the spondylometaphyseal dysplasia Kozlowski type (SMDK). Of all the skeletal dysplasias associated with mutations in the TRPV4 gene, SMDK has the most similar features to MD. Dai et al^[2]^ found that although both disorders have some radiological signs, the presence or absence of a dumbbell-shaped femur is an important feature in distinguishing MD from SMDK.

Exon 15 of the TRPV4 gene is a hotspot for MD-associated variants.^[2]^ While mutations in exon 11 associated with MD are rare, with only 2 mutation sites were reported in a study by Andreucci et al^[19]^ in 2011 as c.1780C > A; p.R594S and c.1781G > A; p.R594H. The mutation site in this study was located in exon 11, c.1811T > A; p. I604N, which had not been reported in previous studies. The patient presented with multiple joint contractures in addition to MD-related clinical phenotypes, combined with progressive hypokinesia of both lower extremities, and no significant compression was seen on spinal MRI.

Mutations in the TRPV4 gene can cause both skeletal and neurological phenotypes. Unger et al^[20]^ described patients with MD with a neuromuscular pathology phenotype who presented with multiple joint contractures and limb dyskinesias, a normal neurological examination, but movement limitations, lighter in the upper limbs, and signs of a chronic axonal denervation process, which is in line with our case.

Skeletal dysplasia does not usually cause multiple joint flexures, and multiple congenital joint contractures represent an overlap between the neuromuscular and skeletal phenotypes of autosomal dominant TRPV4 disorders.^[20]^ It might be difficult to differentiate between neurological symptoms caused by TRPV4 variations in patients with severe skeletal dysplasia because severe skeletal manifestations can compress nerves and result in neurological symptoms. The present patient presented with reduced muscle strength in the distal extremities, which is inconsistent with spinal cord compression in scoliosis, suggesting that this TRPV4 mutation may result in a mixed phenotype of severe skeletal dysplasia and neuromuscular pathology with motor inability.

The prognosis for MD patients varies according to severity. Scoliosis progression is an important factor affecting patients’ quality of life, and our patient underwent orthopedic spinal surgery to delay the course of the disease, and relevant studies have shown that timely surgical intervention can effectively mitigate the progression of scoliosis in the disease.^[21,22]^ According to this study, surgery is superior than kyphosis correction for scoliosis correction and is less likely to reoccur. Bilateral hip dislocation is also a common complication in patients with MD, and conservative treatment seems to provide better outcomes for patients, while surgery is unlikely to improve their prognosis.^[23]^ Of course, surgical osteotomies can be done to correct multiple joint deformities if they have a significant negative impact on the patient’s quality of life. Early professional diagnosis and treatment can reduce the progression of the disease and enhance the patient’s quality of life, but they cannot alter the disease’s natural course.

In conclusion, the present study confirmed the diagnosis of MD based on growth and developmental history, clinical presentation, imaging and mutation analysis of the TRPV4 gene. Furthermore, it reported for the first time that a missense mutation in exon 11 of the TRPV4 gene at position 1881 (c. 1881T > A, p. I604N) resulting in a mixed phenotype of skeletal and neuromuscular pathology, which further complements the spectrum of mutations in the TRPV4 gene and the phenotype of MD. This study also provides a reference for prenatal diagnosis, genetic counseling, mechanistic studies, and development of symptomatic treatment for this type of disease.

There are several limitations to this study; first of all, this study is only a case report, and more clinical research is needed in future studies. Secondly, we did not provide the full clinical photos from the age of the first admission. However, we can also observe the long trunk and the relatively greater increase in length of limbs in comparison to the trunk from the whole spine in anterolateral view and in the anterolateral position of both lower extremities.

## 4. Patient consent

The studies involving human participants were reviewed and approved by the Third Affiliated Hospital of Zhengzhou University. Written informed consent to participate in this study was provided by the participants’ legal guardian/next of kin. Written informed consent was obtained from the minor’s legal guardian for the publication of any potentially identifiable images or data included in this article.

## Author contributions

**Conceptualization:** Yu Liu, Bing Xia, Weiming Hu, Yufeng Zhao.

**Data curation:** Yu Liu, Junfang Xu, Bing Xia, Weiming Hu, Xinwei Li, Feipeng Wang, Yufeng Zhao.

**Formal analysis:** Yu Liu, Yanzhao Dong, Junfang Xu, Guoming Feng.

**Funding acquisition:** Yu Liu, Yanzhao Dong.

**Investigation:** Yu Liu, Junfang Xu, Bing Xia, Feipeng Wang, Guoming Feng.

**Methodology:** Yu Liu, Yanzhao Dong, Weiming Hu.

**Project administration:** Feipeng Wang.

**Resources:** Yanzhao Dong.

**Software:** Feipeng Wang.

**Supervision:** Yanzhao Dong, Weiming Hu.

**Visualization:** Weiming Hu.

**Writing – original draft:** Yu Liu, Weiming Hu.

**Writing – review & editing:** Yu Liu, Yanzhao Dong.
